# Diagnosis and Management of Iridocorneal Endothelial Syndrome

**DOI:** 10.1155/2015/763093

**Published:** 2015-09-16

**Authors:** Marta Sacchetti, Flavio Mantelli, Marco Marenco, Ilaria Macchi, Oriella Ambrosio, Paolo Rama

**Affiliations:** ^1^Cornea and Ocular Surface Unit, Ospedale San Raffaele IRCCS, Via Olgettina 60, 20132 Milan, Italy; ^2^Department of Biology, College of Science and Technology, Temple University, 1900 N. 12 Street, Philadelphia, PA 19122, USA; ^3^Department of Sense Organs, Sapienza University, Viale del Policlinico 155, 00186 Rome, Italy; ^4^Department of Ophthalmology, Campus Bio-Medico University, Via Alvaro del Portillo 200, 00128 Rome, Italy

## Abstract

The iridocorneal endothelial (ICE) syndrome is a rare ocular disorder that includes a group of conditions characterized by structural and proliferative abnormalities of the corneal endothelium, the anterior chamber angle, and the iris. Common clinical features include corneal edema, secondary glaucoma, iris atrophy, and pupillary anomalies, ranging from distortion to polycoria. The main subtypes of this syndrome are the progressive iris atrophy, the Cogan-Reese syndrome, and the Chandler syndrome. ICE syndrome is usually diagnosed in women in the adult age. Clinical history and complete eye examination including tonometry and gonioscopy are necessary to reach a diagnosis. Imaging techniques, such as in vivo confocal microscopy and ultrasound biomicroscopy, are used to confirm the diagnosis by revealing the presence of “ICE-cells” on the corneal endothelium and the structural changes of the anterior chamber angle. An early diagnosis is helpful to better manage the most challenging complications such as secondary glaucoma and corneal edema. Treatment of ICE-related glaucoma often requires glaucoma filtering surgery with antifibrotic agents and the use of glaucoma drainage implants should be considered early in the management of these patients. Visual impairment and pain associated with corneal edema can be successfully managed with endothelial keratoplasty.

## 1. Introduction

Iridocorneal endothelial (ICE) syndrome is a rare disorder (ORPHA64734 available at http://www.orpha.net/consor/cgi-bin/OC_Exp.php?lng=en&Expert=64734) characterized by proliferative and structural abnormalities of the corneal endothelium, progressive obstruction of the iridocorneal angle, and iris anomalies such as atrophy and hole formation [1]. The consequences of these changes are cornea decompensation and glaucoma, which represent the most frequent causes of visual function loss in patients with ICE syndrome [2]. The ICE syndrome comprises a spectrum of clinical entities: progressive essential iris atrophy, Cogan-Reese syndrome, and Chandler syndrome [3].

In 1903 Harms extensively described a rare ocular condition characterized by iris atrophy and glaucoma, known as “progressive essential iris atrophy” [4, 5]. Five decades later, Chandler described a rare, unilateral ocular condition characterized by iris atrophy associated with corneal endothelial alterations, corneal edema, and glaucoma [6]. Subsequently, it was suggested that this “Chandler syndrome” and the “progressive essential iris atrophy” are two different forms of the same disease [6, 7]. When Cogan and Reese described a similar condition associated with iris nodules, a third clinical entity was identified and subsequently named “iris nevus” or “Cogan-Reese syndrome” [8–10]. Subsequent studies confirmed that these clinical entities show similar history and clinical findings and share the same pathogenic mechanisms characterized by an abnormal proliferation of corneal endothelium and the unifying term of “iridocorneal endothelial syndrome” was suggested by Yanoff [1, 3, 7, 9, 11].

ICE is sporadic in presentation; it is usually unilateral and typically affects adult patients (more often women in the third to fifth decade) and eventually severely compromises the visual function if not properly treated [1]. Even when they are promptly treated, surgical interventions for these conditions have variable success rates and the management of ICE syndrome represents a challenge for ophthalmologists.

## 2. Etiology

The etiology of ICE syndrome is still largely unknown; however, a series of possible triggering events has been described and the debate on ICE syndrome's etiology is still ongoing after more than a century.

Inflammation in patients with the ICE syndrome was mentioned in few early reports and more than one author described the onset of uveitis in these patients [10, 12]. Scheie and Yanoff reported clumps of chronic inflammatory cells in the iris and vitreous in one eye examined histopathologically, and Shields and colleagues observed anterior chamber inflammation in 3 cases [1, 10]. Patel and colleagues also mentioned that an occasional macrophage was observed on the corneal endothelium in 2 cases [13]. Similarly, Eagle Jr. and colleagues described a mild chronic iridocyclitis in 10 out of 16 consecutive patients diagnosed with Cogan-Reese syndrome [3]. This experience is in line with the report of the group of Alvarado, who described 16 out of 25 patients with ICE syndrome with a red eye or a mild uveitis before disease onset and also documented photographically the presence of keratic precipitates in one of these patients [12].

It was the group of Alvarado that first postulated that the endotheliopathy responsible for the development of this syndrome could have a viral origin [12]. In fact, they noted that the endothelial alterations observed in ICE syndrome patients are similar to those observed in viral disorders. In line with this hypothesis, ICE syndrome diseases are usually monolateral acquired disorders, suggesting that affected patients had one eye primarily affected with a virus during the postnatal age and the other eye protected by immune surveillance established a few weeks after the first infection. The seldom described bilateral occurrence of ICE syndrome could be explained by the simultaneous infection of both eyes [12]. In addition, HSV-DNA was detected in the aqueous humor of patients with idiopathic corneal endotheliopathy suggesting a viral origin of these disorders. Nevertheless a direct proof of a relationship between ICE syndrome and herpes simplex keratitis occurring in the same eye has not been demonstrated [12, 14]. However, the first eight years of studies and experiments by using ultrastructural methods and viral cultures to confirm the hypothesis of a viral origin for the ICE syndrome was a total failure. Nevertheless, they did not lose faith in their hypothesis and later decided to use a new method that seemed advantageous for the detection of viral DNA: the polymerase chain reaction (PCR). This “new” technique finally allowed them to detect herpes simplex virus- (HSV-) DNA in corneal tissue and aqueous humor samples from patients with ICE syndrome [12]. Specifically, the authors found HSV-DNA in more than 60% of the tested samples. They also evaluated the possible presence of different viruses' DNA to explain the HSV-negative samples; however, both herpes zoster and Epstein-Barr viruses were not detected. To further prove their results, the authors also performed PCR to detect viral DNA in samples from healthy subjects and from patients affected by different corneal disorders, including bullous keratopathy and keratoconus, and all were negative. It is interesting to underline that the authors also performed the PCR test on the unaffected eye of a patient with ICE syndrome that was also negative [12]. Other authors have shown that Epstein-Barr virus may also play a role in the disease development [15].

The pioneering work of Alvarado and colleagues strongly suggests that HSV may have a relevant etiologic role in the development of ICE syndrome. However, it may not be the only cause or predisposing factor and a lot has yet to be learned on the disease etiology, which remains partially unknown today.

## 3. Pathogenesis

The pathogenic mechanisms behind the clinical alterations observed in ICE syndrome have been identified in an abnormal proliferation of the corneal endothelium [16–18].

Corneal endothelium is a single layer of uniform, hexagonal cells localized at the inner surface of the cornea into the anterior chamber of the eye. The corneal endothelium lays on a basement membrane, the Descemet membrane. Corneal endothelial cells have an embryological derivation from neural crests. In postnatal age they are postmitotic and, in normal conditions, do not divide. In adult age, corneal endothelial cell density is approximately 3000 cells/mm² and it slightly decreases with age [19]. The function of corneal endothelium is to actively maintain corneal transparency through the regulation of fluid, nutrients, and solute transportation between aqueous humor and the cornea structures [20].

In 1978 Campbell and colleagues proposed the “membrane theory” to explain the pathogenesis of ICE syndrome. Specifically, they hypothesized that in the ICE syndrome corneal endothelial cells are primarily affected and show proliferative and structural abnormalities and the ability to migrate into the surrounding tissues [7]. This hypothesis was supported by the evidence obtained by studies with specular microscopy that showed morphologic changes in size and shape of endothelial cells, resembling epithelial cells, also at the earliest stages of all ICE syndromes [16, 17, 21–23]. Moreover, histopathologic studies of eyes with ICE syndrome showed altered corneal endothelial cells with morphological characteristics resembling an epithelial-like phenotype, named “ICE-cells” in 1985 by Sherrard and colleagues [17, 18]. The other observations supporting this hypothesis derive from histologic studies that demonstrated the presence of a membrane composed of endothelial-like cells with a basement membrane obstructing the anterior chamber angle and covering the iris [1, 24].

It was through transmission electron microscopy studies that it was finally confirmed that the endothelial cells of affected patients are abnormal as they develop unique characteristics of epithelial cells [25]. Specifically, electron microscopic examination of these cells has evidenced desmosomes, intracytoplasmic filaments, filopodia, and microvilli [12, 13, 25, 26]. It is worthy of note that corneal edema observed in patients with ICE syndrome had been explained, before these electron microscopy studies, only by a reduction in number of endothelial cells. However, we now know that this is not the case, as the corneal edema is rather caused by the altered endothelial cell function caused by multiple abnormalities of the endothelial cell barrier. In line with the hypothesis of an abnormal endothelial function rather than a reduced number of endothelial cells, Bourne and Brubaker have also shown that before chronic edema develops, the endothelial barrier is actually more impermeable than in healthy subjects [27]. This observation also correlates very well with the hypothesis that the whole disease pathogenesis may be related to reparative activities induced by the injury of endothelial cells caused by a viral infection or by inflammation. In fact, the formation of microvilli and filopodia is well known during the wound-healing process in animal models of endothelial damage as well as in humans whose endothelial cells have been inadvertently damaged by argon or YAG laser [28]. Thus, the presence of these endothelial abnormalities in ICE syndrome may simply reflect the fact that the endothelium is engaged in reparative activities. Unfortunately, however, this activation is later followed by cell damage and loss of function, necrosis, and a continued decrease in cell density, which may be explained, once again, by a viral/inflammatory etiology of the disease [29].

Immunohistochemistry studies showed the presence of vimentin and cytokeratins (CK) in ICE-cells [18, 30, 31]. Levy et al. demonstrated that ICE-cells expressed a profile of differentiation markers (CK5 and CK19, but not CK3, CK8, and CK18) that resembles that of normal limbal epithelial cells suggesting that ICE syndrome may result from ectopic embryonic ocular surface epithelium [32]. Alternatively, these findings are consistent with a metaplastic stimulus resulting in a profound change in the phenotype of normal corneal endothelial cells [31, 32].

Regardless of the etiologic trigger, the final result of all these cellular alterations is that the abnormal endothelial cells in ICE syndrome migrate posteriorly beyond the Schwalbe line to obstruct the iridocorneal angle and into the anterior chamber to cover the iris, where they form an abnormal basement membrane that eventually contracts triggering pupil shape anomalies, iris atrophic damage, and formation of synechiae between adjacent structures [7].

The angle obstruction also causes an increase of intraocular pressure (IOP) and consequent development of glaucoma in 46% to 82% of patients with ICE syndrome [2].

## 4. Clinical Presentation

ICE syndrome is usually diagnosed in young adults, most often females, although few cases have been described with early onset in children [33–35].

Patients usually present to the ophthalmologist for a change in the shape or position of the pupil. In other cases, patients refer impairment of visual function that ranges from worsening of visual function in the morning due to the corneal decompensation at early stage to blurred vision and/or halos around lights due to glaucoma to a constant reduction in visual acuity. Alternatively, the first diagnosis of an ICE syndrome is made during a routine ocular examination, following the visualization of the abnormal corneal endothelium and/or following the evaluation of the anterior chamber angle by gonioscopy during clinical investigations for a glaucoma suspect.

Although clinical characteristics of ICE syndrome may aid a correct diagnosis, in some cases with severe corneal edema diagnosis may be difficult [2, 36]. This may be due to difficult visualization of the anterior chamber structures when obscured by the edema.

When the corneal endothelial function is sufficient to guarantee corneal transparency, a careful examination of the corneal endothelium can aid towards the diagnosis of an ICE syndrome: a high magnification slit-lamp examination can show a fine, “hammered-silver” or “beaten-bronze” appearance of the endothelium similar to that typically observed in Fuchs dystrophy (FECD). Changes of corneal endothelium in ICE syndrome may be visualized and further evaluated by specular microscopy and, more recently, by in vivo corneal confocal microscopy [17, 37]. Demonstration of the presence of the “ICE-cells” at specular microscopy allows confirming the diagnosis of ICE syndrome. These ICE-cells are typically abnormal, rounded, large, and pleomorphic, with specular reflex showing a typical “light-dark reversal” consisting in a dark surface, with occasional central light spot, and intercellular light borders [17, 21, 23]. The four morphological appearances of these cells described by Sherrard et al. coexist with other cell types giving rise to the four basic ICE variants: (i) disseminated ICE, with ICE-cells scattered throughout an endothelium that appears otherwise essentially normal; (ii) total ICE, with ICE-cells totally replacing the normal endothelium; (iii) subtotal ICE(+), with ICE-cells replacing a variable portion of the endothelium and the remaining being composed of very small cells; and (iv) subtotal ICE(−), with ICE-cells replacing a variable portion of the endothelium and the remaining being composed of enlarged cells [17].

In vivo confocal microscopy (IVCM) is a noninvasive, high resolution imaging technique that represents a useful diagnostic tool in ICE syndrome, also in patients with corneal edema.

It allows the study of all corneal structures at cellular level, providing in vivo images of all corneal cells layers comparable to ex vivo histochemical techniques. In vivo confocal microscopy in patients with ICE syndrome will reveal the presence of “ICE-cells” as pleomorphic epithelial-like endothelial cells with hyperreflective nuclei and cell boarders appearing brighter than cell surfaces [38]. Different “epithelial-like” presentations of endothelial cells have been described at IVCM: one type of abnormal endothelium with quite regular size and shape, a second cell type more irregular in size and shape, similar to the epithelial wing cells on IVCM, and a third, highly irregular cell pattern resembling a surface corneal epithelium. It has been hypothesized that these different observations may be related to the stage of the disease [21, 39, 40].

Nevertheless, although the visualization of these cells can allow making a diagnosis, it is usually considered mandatory to confirm the clinical diagnosis by gonioscopy: in fact, the anterior chamber angle abnormalities are common to all the ICE syndrome subtypes and include broad-based iridotrabecular synechiae that gradually progress until a complete angle closure develops, if not properly treated. While the visualization of these angle alterations is not generally difficult, it must be kept in mind that the membrane obstructing the trabecular meshwork may be initially difficult to visualize by gonioscopy, and the patients' condition may be confused with a more common open-angle glaucoma. This is especially true if corneal edema is believed to be secondary to the intraocular pressure increase rather than to endothelial pump function insufficiency.

The use of ultrasound biomicroscopy (UBM) may represent a useful tool for the detection of changes of the anterior chamber angle structures in ICE syndrome, especially in the presence of corneal edema that does not allow gonioscopy visualization [41]. In addition, combining UBM with gonioscopy evaluation of peripheral anterior synechiae (PAS) may allow a better characterization of the extent (gonioscopy) and shape (UBM) of PAS in ICE syndrome. Zhang and colleagues reported UBM analysis of 21 eyes with ICE syndrome and observed the presence of PAS in all patients associated with a decrease of anterior chamber depth when compared to normal subjects. The authors demonstrated that UBM was more effective in revealing both PAS and iris atrophy than clinical evaluation at slit-lamp biomicroscopy and gonioscopy alone. Four patients also showed angle closure in the fellow eye at UBM examination. In addition, UBM may identify specific features of the different clinical forms of ICE syndrome [42]. Specifically, in patients with progressive iris atrophy, UBM showed marked iris atrophy, and PAS were less pronounced than in patients with Cogan-Reese syndrome. UBM in Chandler syndrome showed the presence of marked corneal edema with Descemet's folds while PAS were less evident. Patients with Cogan-Reese syndrome showed more extensive, often “arborized” PAS. The more severe extent and height of PAS observed by UBM in progressive iris atrophy and Cogan-Reese syndrome support the evidence of a more severe glaucoma observed in these ICE subtypes than in Chandler's syndrome [1, 42].

In any event, a strict follow-up for glaucoma must always be performed in patients with ICE syndrome, by periodical measuring of intraocular pressure, gonioscopy, and visual field and retina examinations [43, 44].

As previously mentioned, once an ICE syndrome diagnosis is made based on these common clinical features, there are at least three different clinical subtypes of the ICE syndrome (Table 1). Wilson and Shields described a series of 37 patients with ICE syndrome showing essential iris atrophy (22%), Chandler syndrome (57%), and Cogan-Reese syndrome (22%) to characterize this condition and describe the clinical course over 12 years [35]. Most of the clinical differences that allow making a differential diagnosis between them are based on the different level of iris involvement and type/severity of iris abnormalities (Table 1).

**Figure 1:**
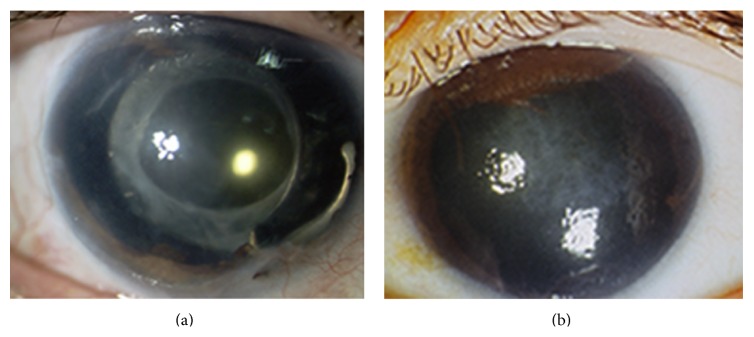
Two patients with essential iris atrophy showing extensive iris atrophy and peripheral anterior synechiae (a, b) and corneal edema (b).

Progressive iris atrophy is characterized by marked iris atrophy and hole(s) formation, which can be of two distinct subtypes: stretch holes, resulting from iris thinning on the other side of the direction of pupillary distortion, and melting holes, in which the iris tissue vanishes without previous signs due to tissue ischemia (Figure 1). Gonioscopy may show the presence of PAS causing variable degrees of angle closure and consequent intraocular pressure increase.

**Figure 2:**
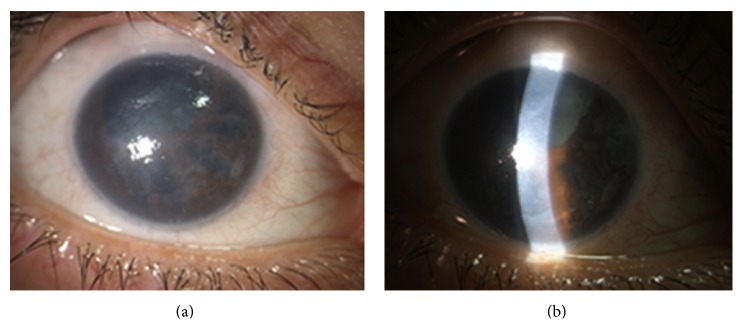
A patient with Chandler syndrome and glaucoma in her right eye showing moderate corneal edema (a), polycoria (b), and peripheral anterior synechiae. She underwent trabeculectomy 7 years earlier. Visual acuity in the right eye was 0.2 decimal units.

In Chandler syndrome, the iris alterations are minimal and the disease is more often diagnosed at early stage by clinicians starting from the observation of a corneal edema. When diagnosis is late and iris anomalies are more pronounced, areas of iris atrophy can be observed but usually never lead to a full-thickness iris hole (Figure 2). Glaucoma may develop due to angle obstruction and PAS.

Finally, in Cogan-Reese syndrome different degrees of iris atrophy can be observed; however the diagnosis is usually made following the observation of a different feature: the presence of multiple iris nodules, usually pedunculated, surrounded by stromal iris showing loss of crypts and a matted appearance. Iris nodules in Cogan-Reese syndrome may develop late in the course of the disease, they appear as fine, yellowish nodules on the iris surface, and later in the disease course they become brown and increase in number.

Although from all the above it may seem that the diagnosis of the different ICE syndrome is quite straightforward, in clinical practice the progressive course of the disease often leads to a challenging diagnosis with a large percentage of cases appearing as mixed forms in the different stages of the disease. As it will be discussed later in detail, the clinical management including surgical approach is usually not linked to the exact diagnosis of the clinical subtype but to the degree of complications such as corneal edema or glaucoma.

Finally, since bilateral cases of ICE syndrome have been reported and subclinical changes have been also described in the contralateral unaffected eye, a careful examination of the fellow eye should be performed, including slit-lamp evaluation of the anterior segment structures, gonioscopy, tonometry, and endothelium evaluation by specular and/or in vivo confocal microscopy [45–48].

## 5. Differential Diagnosis

ICE syndrome should be considered in the differential diagnosis for any young adult (especially women) presenting with unilateral iris anomalies, glaucoma, and/or corneal edema [1]. In fact, although the aspect of the ICE syndrome is characteristic, there are different anterior segment diseases that may mimic it and that may be complicated by the same issues such as corneal edema and glaucoma. Among them, corneal endothelial disorders such as posterior polymorphous dystrophy (PPCD) and Fuchs endothelial dystrophy and iris disorders such as Axenfeld-Rieger syndrome, iris melanoma or inflammatory iris nodules, and aniridia should be considered in the differential diagnosis.

Specifically, endothelial cells in PPCD and FECD show epithelial-like changes and express cytokeratins similarly to ICE syndrome [49, 50]. Among them, the FECD is the easiest to diagnose as this disease features similar (but coarser) endothelial anomalies in both eyes and it does not show the anterior chamber, iridocorneal angle, or iris changes always seen in ICE syndrome. Differential diagnosis may be easily reached by IVCM, which will identify the presence of the “ICE-cells” on the corneal endothelium confirming the diagnosis of ICE syndrome [51].

Patients with PPCD, on the other hand, can have several features that strictly resemble the ICE syndrome, making the differential diagnosis more challenging: in fact, in PPCD endothelial metaplasia, pupil abnormalities, iris alterations, corneal edema, and glaucoma caused by angle closure can be observed [49]. Usually the natural history of the disease and a confocal microscopy evaluation can help in making the proper diagnosis, since, instead of the typical “ICE-cells,” in PPCD a mixed range of endothelial vesicles and bands are observed. However, probably the most compelling difference with ICE syndrome is that PPCD is bilateral, and patients also have a typical familiar history since it is an autosomal dominant disease [52].

Another disease that may be very challenging to distinguish from an ICE syndrome is the Axenfeld-Rieger syndrome. In fact, even the pathogenesis of this syndrome is similar as the iris and iridocorneal angle alterations seen in Axenfeld-Rieger syndrome are also caused by a layer of endothelial cells. The only difference is that in Axenfeld-Rieger syndrome they are not secondary to the migration but rather to the presence of a primordial endothelial layer. From a clinical point of view, therefore, the only difference between the two syndromes is that in Axenfeld-Rieger the findings are bilateral and congenital and are often stationary or only have a minor progression over time. Also, at confocal microscopy the corneal endothelium in these patients does not appear altered [53].

Lastly, since in aniridia there are often rudimentary iris stumps and not a complete absence of the iris, this disease may be confused with a late stage progressive iris atrophy. In fact, both can be complicated by glaucoma and corneal clouding. However, in aniridia the corneal clouding is usually caused by a pannus due to a limbal stem cell deficiency and not by endothelial cell dysfunction. In addition, aniridia is a bilateral congenital disease caused by a defect in the PAX6 gene; therefore, additional congenital ocular malformations are often present, including optic nerve hypoplasia, and patients usually have very poor vision and nystagmus [54].

Iris nodules observed in Cogan-Reese syndrome also require differential diagnosis with other conditions showing similar iris changes such as neurofibromatosis, iris melanoma, and sarcoidosis. Von Recklinghausen's neurofibromatosis shows pigmented iris nodules which, differently from Cogan-Reese syndrome's nodules, are bilateral, flatter, and more similar to iris nevi [55]. The presence of intraocular inflammation may help differentiate iris nodules in sarcoidosis form that present in Cogan-Reese syndrome.

Another important differential diagnosis of iris nodules in Cogan-Reese syndrome is the malignant melanoma of the iris. The presence of endothelial changes and cornea edema, PAS, and iris atrophy may correctly orient the diagnosis toward the ICE syndrome [56].

## 6. Management and Treatment

The HSV hypothesis in the pathogenesis of the ICE syndrome suggests that antiviral treatment may be beneficial in the management of this disease [12]. However, this has yet to be proven and, to date, there is no medical or surgical treatment that can definitely solve any of the ICE syndrome subtypes, and the final therapeutic target is the prevention and management of the visual impairing complications, namely, corneal edema and glaucoma.

The use of hypertonic saline solution instilled as eye drops may be beneficial in the morning to reduce the corneal edema when it is more pronounced.

Topical antiglaucomatous medications are usually the first line of treatment, since a reduction in intraocular pressure can also improve corneal edema. Suppressants of aqueous humor production, including topical beta blockers, alpha agonists, and carbonic anhydrase inhibitors, are preferred and are usually accompanied by miotics although their added value is considered minimal [57]. Since the role of HSV in ICE syndromes has not been completely ruled out, prostaglandins should be used with caution in patients with ICE-related glaucoma as their use has been reported to stimulate recurrence of herpes simplex [58]. High failure rate (from 60% to 88%) of medical treatment for glaucoma is reported in literature and when topical treatments fail or are insufficient, surgical approaches are followed [2, 11]. In a case series of 82 consecutive cases, 37 (45%) required one or more trabeculectomies [59]. Filtrating surgery in ICE-related glaucoma showed a lower success rate than in other types of glaucoma [2, 59, 60]. Studies showed a survival rate of trabeculectomy of about 60% after 1 year and 40% after 2 years of follow-up [59]. These percentages decrease for reinterventions below 20% of success rate [2]. Antifibrotic agents have been proposed to increase the success rate of filtrating surgery in ICE syndrome. The use of postoperative 5-fluorouracile showed failure in 5 out of 9 patients that required additional glaucoma surgery within 1 year [61]. Intraoperative use of mitomycin-C in 10 patients with ICE syndrome and glaucoma showed a good IOP control in 8 out of 10 eyes after a mean of 14.9 months of follow-up [60]. A larger study on 26 patients with ICE-related glaucoma showed a survival rate of trabeculectomy with antifibrotic agents of 73% at 1 year and 44% and 29% after 3 and 5 years [62]. This study also reported that needle and manipulation of the bleb and trabecular flap in these patients did not increase the success rate [62]. The failure of filtering surgery may be due to the progressive growth of the abnormal endothelial membrane extending over the trabecular meshwork and the filtration site [3, 13]. By this point of view, glaucoma drainage implants (GDIs) may overcome the regrowth of the membrane in the filtration site, and studies on the efficacy of GDIs in ICE-related glaucoma showed high success rate of about 70% at 1 year, form 70% to 40% after 3 years, and of 53% after 5 years [62, 63]. In these studies, 20%–50% of patients required replacement or repositioning of the tube, and the authors advise lengthening the tube allowing the possibility of future reposition and keeping the tip of the tube far away from cornea and iris structures [62].

Some studies reported a higher success rate of glaucoma management and surgery in patients with Chandler syndrome, and the authors hypothesize that this different clinical outcome may be due to the less aggressive proliferative endothelial growth observed in these patients [35, 59, 62]. Regardless of the type of surgical approach, cyclodestructive procedures such as cyclophotocoagulation are still very often needed because intraocular pressure control is very challenging in ICE syndrome patients, who are younger than the typical glaucoma patient and, therefore, tend to have a more pronounced cicatrizing response that can lead to the failure of all filtering procedures [2].

In advanced cases of corneal edema with well-controlled IOP, corneal surgery should be considered to improve visual function and reduce pain. Penetrating keratoplasty (PK) was proposed in few small reports with short follow-up, with variable success rate from 83% to 100% [64–67]. Penetrating keratoplasty was able in improving visual function and pain relief in patients with ICE syndrome; it also allowed a clear media for monitoring of optic disc and visual field changes in patients with associated glaucoma. Long-term results from DeBroff and Thoft reported graft failure in 83% and graft rejection in 2 out of the 6 eyes of patients with essential iris atrophy treated with PK. They also reported the presence of postoperative anterior uveitis resistant to corticosteroid treatment in all eyes [68]. Alvim and colleagues revised the surgical outcome in 14 patients with ICE syndrome followed up to 58 months after PK. They reported early graft failure in 50% of patients due to rejection in 6 patients and endothelial failure in 1 patient. At the end of follow-up, clear graft was reported in 85% of cases, with 6 patients requiring a second PK [69].

In 2007, M. O. Price and F. W. Price Jr. reported the successful use of Descemet stripping with endothelial keratoplasty (DSEK) in 3 pseudophakic patients with ICE syndrome and corneal edema, introducing endothelial surgery in the surgical treatment of ICE syndrome [70]. Endothelial keratoplasty is a surgical procedure that selectively replaces dysfunctional endothelium, sparing the corneal stroma and epithelium. This surgical technique offers several advantages for the treatment of corneal edema in ICE syndrome, when compared with PK. In fact, endothelial keratoplasty provides rapid visual recovery with minimal refractive changes, avoids the use of sutures, and better maintains corneal recipient integrity and innervation [71]. Both deep lamellar endothelial keratoplasty (DLEK) and the Descemet stripping endothelial keratoplasty (DSEK) have been successfully performed in patients with ICE syndrome [70, 72, 73]. The DSEK procedure consists in the replacement of abnormal endothelium and Descemet membrane, while DLEK requires an excision of a posterior lamella of the recipient's cornea stroma. DSEK is simpler and less invasive and allows more rapid visual function recovery compared to DLEK [74]. However, DLEK may offer some advantages in eyes with ICE syndrome characterized by iris anomalies, PAS, and flatter anterior chamber. In a case series of 7 phakic eyes with ICE syndrome, DLEK was successfully performed by Huang and colleagues, who preferred DLEK because the excision of the recipient bed allowed an easier positioning of the donor graft with less manipulation [75]. Recently, a new endothelial keratoplasty, the Descemet's membrane endothelial keratoplasty (DMEK), has been introduced to achieve better visual recovery and to decrease immunologic rejection [76, 77]. However, the efficacy of this surgical approach in patients with complex anterior ocular segment disorders, such as ICE syndrome, has not yet been demonstrated.

Obviously, since all corneal surgery procedures do not completely remove the abnormal endothelium, they are not able to halt the progression of PAS and glaucoma in ICE patients [66].

Lastly, it is worthy of note that iris reconstruction with or without the use of intraocular prosthesis has been proposed in ICE syndrome for both cosmetic reasons and for reducing the visual disturbances of polycoria [78].

## Figures and Tables

**Table 1:** Clinical features of the different ICE syndrome subtypes.

	Iris	Pupil	Cornea	Anterior chamber angle
Chandler syndrome	Areas of atrophy (not full-thickness holes)	Corectopia	Early and marked edema, endothelial dystrophy, and ICE-cells at confocal microscopy	Peripheral anterior synechiae

Progressive iris atrophy	Full-thickness hole(s)	Polycoria	Endothelial dystrophy, ICE-cells at confocal microscopy, and corneal edema may occur	Peripheral anterior synechiae

Cogan-Reese syndrome	Nodules and iris atrophy	Changes uncommon	Endothelial dystrophy, ICE-cells at confocal microscopy, and corneal edema may occur	Peripheral anterior synechiae
